# Matrix stiffness regulates the triad communication of adipocytes/macrophages/endothelial cells through CXCL13

**DOI:** 10.1016/j.jlr.2024.100620

**Published:** 2024-08-14

**Authors:** Arthur Choisez, Seiichiro Ishihara, Takuro Ishii, Yidan Xu, Sepideh D. Firouzjah, Hisashi Haga, Ryoichi Nagatomi, Joji Kusuyama

**Affiliations:** 1 Department of Biosignals and Inheritance, Graduate School of Medical and Dental Sciences, Tokyo Medical and Dental University (TMDU), Tokyo, Japan; 2 Division of Biomedical Engineering for Health and Welfare, Graduate School of Biomedical Engineering, Tohoku University, Sendai, Japan; 3 Department of Advanced Transdisciplinary Sciences, Faculty of Advanced Life Science, Hokkaido University, Sapporo, Japan; 4 Frontier Research Institute for Interdisciplinary Sciences, Tohoku University, Sendai, Japan; 5 Department of Medicine and Science in Sports and Exercise, Tohoku University School of Medicine, Sendai, Japan

**Keywords:** mechanotransduction, chemokine, genipin, collagen, differentiation

## Abstract

Adipose tissue remodeling and plasticity are dynamically regulated by the coordinated functions of adipocytes, macrophages, and endothelial cells and extracellular matrix (ECM) that provides stiffness networks in adipose tissue component cells. Inflammation and fibrosis are crucial exogenous factors that dysregulate adipose tissue functions and drastically change the mechanical properties of the ECM. Therefore, communication among the ECM and adipose tissue component cells is necessary to understand the multifaceted functions of adipose tissues. To obtain in vivo stiffness, we used genipin as a crosslinker for collagen gels. Meanwhile, we isolated primary adipocytes, macrophages, and endothelial cells from C57BL/6J mice and incubated these cells in the differentiation media on temperature-responsive culture dishes. After the differentiation, these cell sheets were transferred onto genipin-crosslinked collagen gels with varying matrix stiffness. We found that inflammatory gene expressions were induced by hard matrix, whereas antiinflammatory gene expressions were promoted by soft matrix in all three types of cells. Interestingly, the coculture experiments of adipocytes, macrophages, and endothelial cells showed that the effects of soft or hard matrix stiffness stimulation on adipocytes were transmitted to the distant adipose tissue component cells, altering their gene expression profiles under normal matrix conditions. Finally, we identified that a hard matrix induces the secretion of CXCL13 from adipocytes, and CXCL13 is one of the important transmitters for stiffness communication with macrophages and endothelial cells. These findings provide insight into the mechanotransmission into distant cells and the application of stiffness control for chronic inflammation in adipose tissues with metabolic dysregulation.

Extracellular mechanical properties are essential regulators of cellular physiological events including differentiation, migration, proliferation, secretion, apoptosis, and morphogenesis (1). The effects of extracellular matrixes (ECMs) on various cell behaviors are induced by intracellular signaling pathways called mechanotransduction. Previous studies showed that cells sense substrate stiffness via rearrangement and clustering of integrin (2) and mechanosensitive ion channels (3) regulating the roles of transcription factors and the significant changes in gene expression (4). Tissues consist of several types of cells and various ECMs to build up tissue mechanics. Recent findings pointed out that tissue deformation and tissue geometry directly regulate the chromatin responses and the activities of mechanosensitive transcription factors (5). Therefore, only cell-intrinsic responses to matrix stiffness are insufficient to understand the effects of matrix stiffness on tissue physiology and cell-ECM mechanotransduction.

Adipose tissues dynamically undergo remodeling in response to multiple factors such as nutrition, exercise stimuli, and aging. This plasticity of adipose tissues depends on the coordinated functions of adipose tissue component cells including adipocytes, macrophages, endothelial cells, neuronal cells, and fibroblasts (6). Additionally, the ECM is a critical component of adipose tissues, providing mechanical and chemical networks of cells and cell-derived secretory factors (7). Inflammation and fibrosis, major characteristics of metabolically unhealthy adipose tissues, are also caused by both macrophage infiltration and dysregulated ECM characterized as excessive deposition, cross-linking, and unbalanced cleavage or degradation (7, 8). A previous study reported that obesity-induced ECM remodeling of adipose tissue not only changes the structural and biochemical support of adipose tissue cells but also drives obesity-associated cancer progression (9). Therefore, the coordinated communications among different types of cells and ECM characterize the broad effects of adipose tissue properties on whole-body pathogenic status.

ECM remodeling occurs through the alterations of stiffness and elasticity (10). Metabolic dysfunctions such as obesity and type 2 diabetes are associated with mechanical properties of adipose tissue. For example, previous studies reported obesity-induced increases in adipose tissue stiffness in mouse epididymal fat pads (11), mouse mammary fat pads (12), and human adipose tissue (13, 14). A recent study showed that the stiffness of adipocytes is decreased by adipogenic differentiation of preadipocytes, lipid droplet accumulation, and in vivo high-fat diet-induced obesity; however, the adipose tissue from db/db mouse, an obesity-induced type 2 diabetes model, showed the increase of stiffness compared to lean control (15). These reports suggest that adipose tissue stiffness should be explained not only by the mechanical properties of adipocytes but also by the changes in ECM properties and the effects of ECM on adipocyte component cells.

Here, we found that the stiffness of the cell culture matrix affected the expression of cell differentiation, proinflammation-, and antiinflammation-related genes in the adipose tissue component cells including adipocytes, macrophages, and endothelial cells. Inflammatory gene expressions were induced by hard matrixes, whereas antiinflammatory gene expressions were promoted by soft matrixes in all three types of cells. The induction of inflammatory phenotypes by a hard matrix was also observed in the distant cells that were incubated under a normal matrix. We identified that CXCL13 is one of the adipocyte-derived proteins that transmits the hard matrix information to macrophages and endothelial cells. Anti-CXCL13 neutralizing antibodies blocked the hard matrix-induced inflammatory gene expression in the coculture of adipocytes, macrophages, and endothelial cells.

## Materials and Methods

### Primary cell isolation and culture

Primary adipocyte-progenitor cells were isolated from the subcutaneous (anterior parts and posterior parts) and the visceral (perigonadal area and mesenchymal area) adipose tissue of 8–12-week-old C57BL/6J male mice as previously described (16), and 1 × 10^5^ cells were initially cultured in D-MEM (049–32645, Fujifilm Wako) containing 10% fetal bovine serum (FBS) (10437028, Gibco), and 100 mg/ml penicillin/streptomycin (168–23191, Fujifilm Wako). At 90% confluency, cells were induced to differentiate in induction media: D-MEM supplemented with 10% FBS, 1% penicillin/streptomycin, 0.5 mM 3-isobutyl-1-methylxanthine (IBMX) (I-5879, Sigma-Aldrich) in 0.5 N KOH, 1 μM dexamethasone (DEXA) (D-1756, Sigma-Aldrich) in ethanol, and 1 μM insulin (I-0516, Sigma-Aldrich), for 2 days. Cells are subsequently cultured in differentiation media: D-MEM supplemented with 10% FBS, 1% penicillin/streptomycin, and 1 μM insulin for 6 days. Totally, 8-days differentiated cells were used as mature adipocytes.

Primary peritoneal macrophages were isolated from C57BL/6 mice as previously described (17) with minor modifications. Briefly, 8-12-week-old male mice were injected intraperitoneally with 2.5 ml of 5% thioglycolate broth (70157, Millipore). Three days later, 5 ml of cold PBS was injected into their peritoneal cavities, and the PBS was aspirated into the syringe to collect thioglycolate-elicited macrophages. Collected PBS was centrifuged at 300 g for 5 min, and the cells were incubated in the culture dish with RPMI-1640 (189–02415, Fujifilm Wako) containing 10% FBS and 100 mg/ml penicillin/streptomycin for 1 h. Subsequently, the bottom of the dish was washed with PBS three times, and the remaining adherent cells were used as macrophages. M1 macrophages were induced to differentiate in the addition of 10 ng/ml lipopolysaccharide (127–05141, Fujifilm Wako) and 50 ng/ml interferon-gamma (IFN-γ) (090–06981, Fujifilm Wako) for 24 h. M2 macrophages were obtained by the stimulation of 20 ng/ml interleukin-4 (IL-4) (090–06621, Fujifilm Wako) for 24 h.

Primary endothelial cells were isolated from the subcutaneous adipose tissue of 8–12-week-old C57BL/6J male mice based on the previous study (18) with modifications. Briefly, subcutaneous adipose tissues with vasculature are placed on cold dissection buffer (2 mM CaCl_2_, 10 mM D (+) glucose, 10 mM Hepes, 1 mM MgCl_2_, 5 mM KCl, 145 mM NaCl, and pH 7.4) and the parenchymal adipose were carefully removed from the artery. The isolated arteries were incubated in 5 ml of the dissociation solution (10 mM D(+) glucose, 10 mM Hepes, 2 mM MgCl2, 56 mM KCl, 55 mM NaCl, 80 mM sodium hydrogen L(+)-glutamate monohydrate, and pH 7.4) containing 0.5 mg/ml dispase (LS02110, Worthington Biochemical) and 0.5 mg/ml elastase (LS002292, Worthington-Biochemical) at 37°C for 1 h with gentle agitation by inversion every 10–15 min. Subsequently, 2.5 mg collagenase type I (LS004194, Worthington Biochemical) was added to the cell suspension and incubated at 37°C for 20 min with gentle agitation. The cell suspension was mechanically dissociated by vigorous trituration with a pipette, filtered through a 70 μm cell strainer, and centrifuged at 300 g for 3 min. Next, the endothelial cells were purified from the dissociated cells by magnetic-activated cell sorting using MACS separator (130-090-976, Milteny Biotec). The cell pellet was resuspended in 80 μl of DPBS/BSA buffer (Dulbecco’s PBS, 0.5% bovine serum albumin, 2 mM EDTA, and pH 7.2) and 20 μl of CD45 MicroBeads (130-052-301, Milteny Biotec) per 10^7^ cells and incubated at 2–8°C for 20 min with rotation. The volume of cell suspension was adjusted to 500 μl for up to 10^7^ cells and was applied onto LS Column (130-042-401, Miltenyi Biotec) in the magnetic field according to the manufacturer's protocol. Flow-through containing unlabeled cells (CD45−cells) was centrifuged at 300 *g* for 3 min and was similarly performed magnetic sorting with CD31 MicroBeads (130-097-418, Miltenyi Biotec). After washing the column with 2 ml of DPBS/BSA buffer twice, the column was removed from the magnetic separator and placed on a collection tube. The magnetically labeled cells (CD31+ cells) were immediately flashed out with 3 ml of DPBS/BSA buffer. The isolated CD45− CD31+ cells (nonhematopoietic, platelet endothelial cell adhesion molecule positive cells) were incubated with Endothelial Cell Growth Kit-VEGF (PCS-100-041, ATCC) and used as endothelial cells.

All animal studies were approved by the Animal Care and Use Committee of Tohoku University (Approved number: 2020-012-02) and conducted following the institutional guidelines.

### Control of matrix stiffness by the genipin-crosslinked collagen gel

To obtain the different stiffness of the cell culture matrix, the collagen gels were cross-linked by genipin as previously described (19) with minor modifications. Genipin premix solution (genipin [078–03021, Fujifilm Wako] in 100 mM Hepes, 2×PBS [-], and pH 7.4) was prepared for ×2 final genipin concentration (0, 0.01, 0.05, 0.1, 0.5, 1, and 10 mM genipin) and mixed with equal volumes of Atelocollagen Acidic Solution (5 mg/ml collagen, IPC-50, KOKEN) by gently pipetting for 1 min on ice. To remove bubbles, the genipin-collagen mixture was quickly centrifuged at 10,000 *g* for 15 s. The mixture was poured onto an iced cell culture dish and incubated at 37°C for 72 h in a humidified incubator with 5% CO_2_. Following the 1 h incubation with PBS (−) for three times, 24 h incubation with D-MEM or RPMI-1640 without FBS, and 24 h incubation with D-MEM or RPMI-1640 containing 10% FBS and 100 mg/ml penicillin/streptomycin in a humidified incubator with 5% CO_2_, the gels were ready to use the cell culture experiments.

### Transferring of cell sheets to gel-coated plates

The differentiation of primary adipo-progenitor cells, M1/M2 polarization of primary macrophages, and the proliferation of primary endothelial cells were induced on UpCell® (CS3007, CellSeed), temperature-responsive cell culture dishes. When the cells reached the required condition, CellShifter™ (CSD001, CellSeed), a supporting material to transfer the cell sheet from the culture dish, was placed onto the surface of the cell sheet, and the culture dish was incubated at 20°C for 7 min. Then, the CellShifter™ attaching the cell sheet was gently placed on the genipin-crosslinked collagen gel in the dish and incubated at 20°C for 1 min. Finally, the dish was incubated with 150 μl of D-MEM or RPMI-1640 containing 10% FBS and 100 mg/ml penicillin/streptomycin at 37°C for 1 h. The cells were detached from the cell and stimulated by the different matrix stiffness under the same differentiation media.

### Connected culture of adipocytes, macrophages, and endothelial cells

The adipocytes, macrophages, or endothelial cells were cocultured in UniWells™ Horizontal Co-Culture Plate (384–14421, Fujifilm Wako) with UniWells™ Filter 0.6 μm (388–14441, Fujifilm Wako) according to the manufacturer’s instruction. UniWells™ was precoated by the genipin-crosslinked collagen gel. The cell culture media was added to 0.5 ml at the single culture condition and 1.5 ml at the coculture condition.

### Stimulation of conditioned media from adipocytes under different stiffness in macrophages and endothelial cells

The differentiated primary adipocytes were cultured on 0.1 or 0.5 mM genipin-crosslinked collagen gels in D-MEM for 24 h. Cell culture media were collected and centrifuged at 10,000 g for 1 min. M1 macrophages and endothelial cells are cultured with 50% conditioned media for 24 h on 0.1 mM genipin-crosslinked gels.

### Realtime quantitative-PCR

Total RNA was isolated with Isogen II (311–07361, Nippon Gene) and reverse-transcribed with iScript™ Reverse Transcription Supermix for RT-qPCR (1708840, Bio-Rad). Each mRNA expression was calculated relative to that of Rpl13a. Primer sequences are in supplemental Table S1.

### Biochemical assays

Secretory proteins in the culture media were analyzed by Mouse Cytokine Antibody Array (ab133995, abcam) targeting 62 proteins. The amounts of inflammatory cytokines in the culture media were analyzed by the Mouse IFN-gamma Quantikine ELISA Kit (MIF00, R&D systems), Mouse TNF-alpha Quantikine ELISA Kit (MTA00B, R&D systems) and Mouse IL-6 Quantikine ELISA Kit (M6000B, R&D systems) and Mouse CXCL13/BLC/BCA-1 Quantikine ELISA Kit (MCX130, R&D systems).

Cytokine neutralization was performed by mouse CCL2/JE/MCP-1 antibody (AF-479-NA, R&D systems), mouse CXCL13/BLC/BCA-1 antibody (AF340, R&D systems), mouse/rat IFN-gamma antibody (AF-585-NA, R&D systems), mouse IL-1beta Antibody (AF-401-NA, R&D systems), and mouse IL-6 antibody (AF-406-NA, R&D systems).

### Oil red O staining

Lipid droplet appearance was determined by oil red O staining as described previously (20).

### Statistical analysis

All data are represented as the means ± SEM. Statistical significance was defined as *P* < 0.05, 0.01, 0.001, or 0.0001 and determined via one- or two-way ANOVA, with Tukey and Bonferroni post hoc analysis.

## Results

### Matrix stiffness affects the expression of inflammatory, antiinflammatory, and adipose tissue remodeling-related genes in primary adipocytes, primary peritoneal macrophages, and primary endothelial cells

**Fig. 1:**
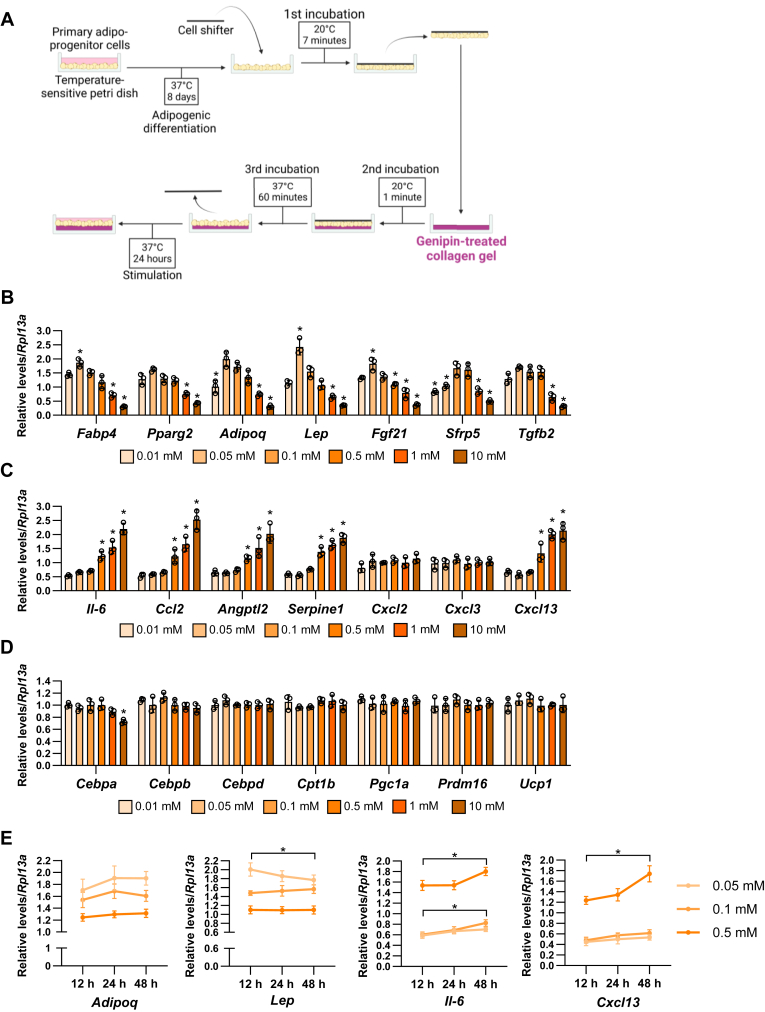
Matrix stiffness affects adipogenic differentiation-related and inflammatory gene expression in primary adipocytes. A: Schematic illustration of the mature adipocyte transferring to the genipin-treated collagen gel. B-D: The mRNA expression of adipogenesis-related (B), inflammatory (C), and adipocyte and beige differentiation-related (D) genes in the different types of matrix stiffness-stimulated adipocytes. (∗*P* < 0.01 vs. 0.1 mM with one-way ANOVA and Tukey and Bonferroni post hoc analysis) E: The mRNA levels of adipocyte gene expressions in 12, 24, and 48 h and on 0.05, 0.1, and 0.5 mM genipin crosslinked gel-stimulated adipocytes. (∗*P* < 0.01 vs. 12 h with two-way ANOVA and Tukey and Bonferroni post hoc analysis).

We induced the differentiation of primary adipocytes from the subcutaneous adipose tissues of 8–12-week-old C57BL/6J male mice in the temperature-sensitive petri dishes. After 8 days, adipocytes were fully differentiated with lipid droplets (supplemental Fig. S1) and the mature adipocytes were transferred into genipin-treated cross-linked collagen gels by cell sheet shifting and cultured on the gel with different stiffness for 24 h (Fig. 1A). The matrix stiffness (Young’s modulus) was controlled by the different concentration of genipin (0 mM: 0.0292 kPa, 0.01 mM: 0.267 kPa, 0.05 mM: 0.678 kPa, 0.1 mM: 1.49 kPa, 0.5 mM: 3.36 kPa, 1 mM: 9.20 kPa, and 10 mM: 12.5 kPa) as reported previously (19). We found that the expressions of adipokines and adipogenesis-related markers, fatty acid binding protein 4 (*Fabp4*), peroxisome proliferator-activated receptor gamma 2 (*Pparg2*), adiponectin (*Adipoq*), and leptin (*Lep*), and fibroblast growth factor 21 (*Fgf21*) were peaked at 0.05 mM genipin-treated collagen gels and dose dependently decreased from 0.1 mM to 10 mM genipin stiffness (Fig. 1B). The mRNA expression of secreted frizzled-related protein 5 (*Sfrp5*) was increased at 0.1 and 0.5 mM genipin and decreased at 10 mM compared to 0.01 mM. The expression of transforming growth factor-beta 2 (*Tgfb2*) was decreased at 1 and 10 mM genipin conditions. On the other hand, the expressions of inflammatory genes such as interleukin-6 (*Il-6*), C-C motif chemokine ligand 2 (*Ccl2*), angiopoietin like protein 2 (*Angptl2*), serpin family E member 1 (*Serpine1*), and C-X-C motif chemokine ligand 13 (*Cxcl13*) were dose dependently increased from 0.1 mM to 10 mM genipin (Fig. 1C). However, the expression of adipogenic differentiation-related chemokines, *Cxcl2* and *Cxcl3* (21), was not affected by matrix stiffness. In other adipogenic differentiation regulators, *CCAAT/enhancer-binding protein alpha* (*Cebpa*) expression was significantly inhibited at 10 mM genipin conditions, however, CEBP beta (*Cebpb*) and CEBP delta (*Cebpd*) expressions and beige markers such as carnitine palmitoyltransferase 1B (*Cpt1b*), PPREg coactivator 1-alpha (*Pgc1a*), PR domain containing 16 (*Prdm16*), and uncoupling protein 1 (*Ucp1*) were not changed by matrix stiffness (Fig. 1D). Moreover, we analyzed the time course effects of matrix stiffness on representative adipokines (Fig. 1E). Promotive effects of 0.05 mM genipin gels on *Lep* were significantly decreased from 12 h to 48 h. In contrast, promotive effects of 0.1 or 0.5 mM genipin gels on Il-6 and Cxcl13 were increased at 48 h stimulation. Collectively, these results suggest that matrix stiffness regulates inflammatory and antiinflammatory cytokines expression in mature adipocytes.

**Fig. 2:**
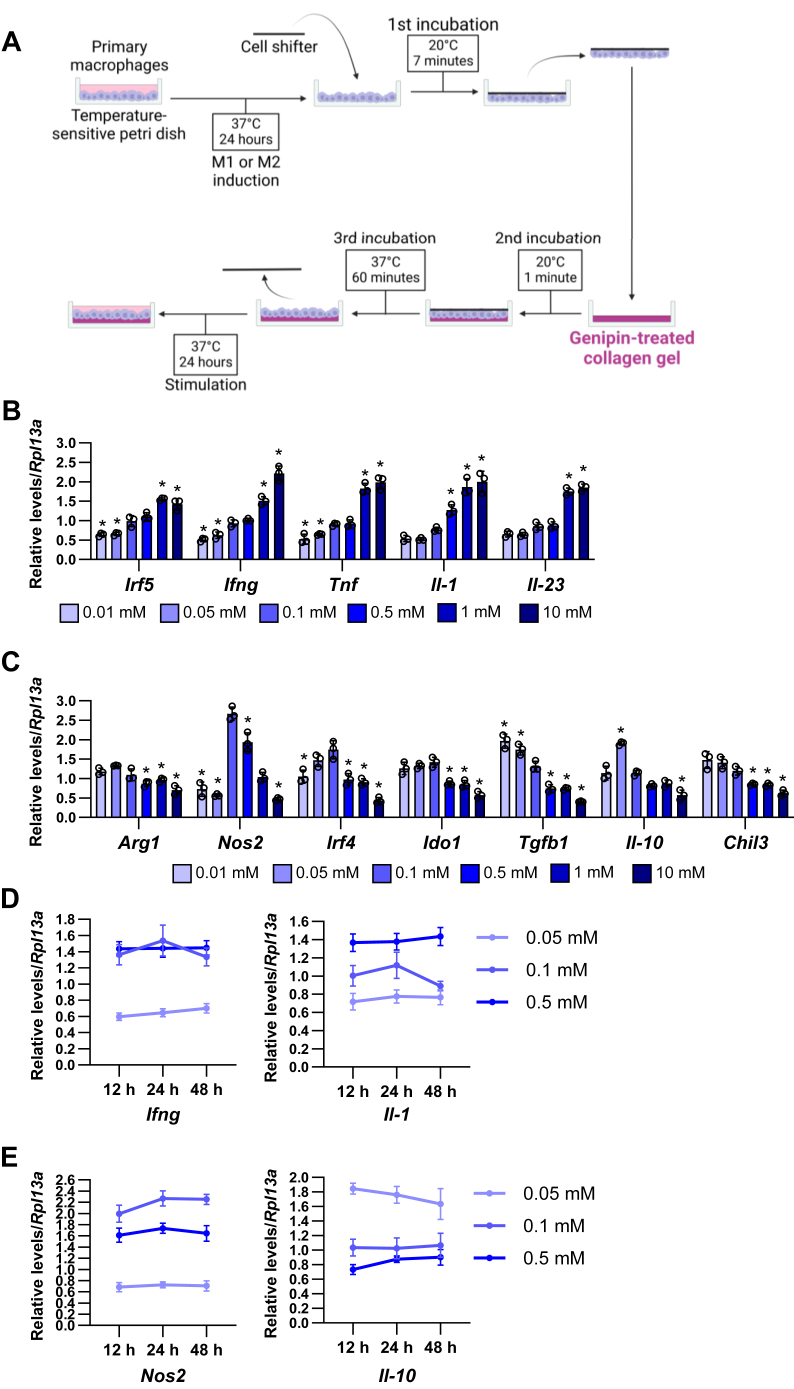
Matrix stiffness affects macrophage differentiation marker expression in primary M1/M2 macrophages. A: Schematic illustration of the M1/M2 polarized macrophage transferring to the genipin-treated collagen gel. B and C: The mRNA expression of M1 (B) and M2 (C) marker genes in the different types of matrix stiffness-stimulated M1 (B) and M2 (C) macrophages. (∗*P* < 0.01 vs. 0.1 mM with one-way ANOVA and Tukey and Bonferroni post hoc analysis). D and E: The mRNA levels of M1 (D) and M2 (E) macrophage genes in 12, 24, and 48 h, and on 0.05, 0.1, and 0.5 mM genipin crosslinked gel-stimulated adipocytes.

Next, we induced the polarization of primary peritoneal macrophages of 8–12-week-old C57BL/6J male mice in the temperature-sensitive petri dishes for 24 h. Then, M1 and M2 macrophages were transferred and cultured on the gel with different stiffness for 24 h (Fig. 2A). The expressions of M1 macrophage-phenotypic genes, interferon regulatory factor 5 (*Irf5*), interferon gamma (*Ifng*), tumor necrosis factor (*Tnf*), *Il-1*, and *Il-23*, were dose dependently increased from 0.1 mM to 10 mM genipin (Fig. 2B). In contrast, the expressions of M2 macrophage-phenotypic genes, arginase-1 *(Arg1*), indoleamine 2,3-dioxygenase 1 (*Iod1*), *Tgfb1*, and chitinase-like 3 (*Chil3*), were dose dependently decreased from 0.1 mM to 10 mM genipin (Fig. 2C). Other types of M2 macrophage genes, nitric oxide synthase 2 (*Nos2*), *Irf4*, and *Il-10*, were peaked at 0.1 mM genipin and dose dependently decreased from 0.1 mM to 10 mM genipin. Time course analysis showed that 12 h–48 h incubation did not affect *Ifng* and *Il-1* in M1 macrophages (Fig. 2D) and *Nos2* and *Il-10* in M2 macrophages (Fig. 2E) on 0.05, 0.1, and 0.5 mM genipin gels. These results indicate the preferable relation of M1 polarization/hard matrix and M2 polarization/soft matrix in the peritoneal macrophages.

**Fig. 3:**
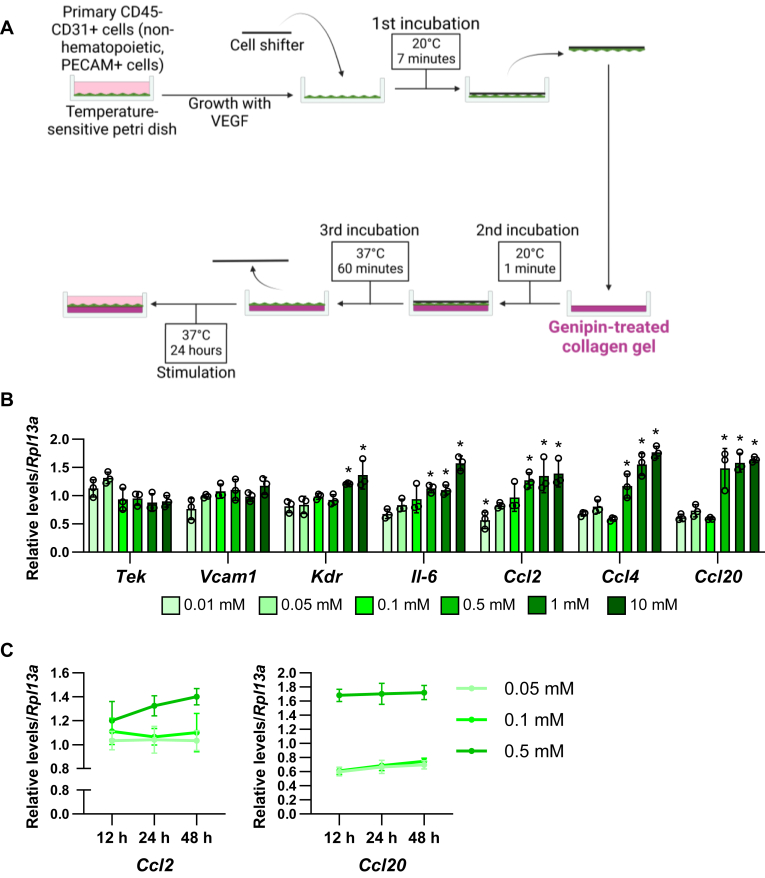
Matrix stiffness affects endothelial marker and inflammatory gene expression in primary M1/M2 macrophages. A: Schematic illustration of the proliferated endothelial cell transferring to the genipin-treated collagen gel. B: The mRNA expression of endothelial marker and inflammatory genes in the different types of matrix stiffness-stimulated endothelial cells. (∗*P* < 0.01 vs. 0.1 mM with one-way ANOVA and Tukey and Bonferroni post hoc analysis). C: The mRNA levels of endothelial cell gene expressions in 12, 24, and 48 h, and on 0.05, 0.1, and 0.5 mM genipin crosslinked gel-stimulated adipocytes.

We also analyzed the effects of matrix stiffness on the primary endothelial cells, CD45−CD31+ cells (nonhematopoietic, platelet endothelial cell adhesion molecule positive cells, which are isolated from the subcutaneous adipose tissues (Fig. 3A)). The expression of endothelial maker genes, TEK receptor tyrosine kinase (*Tek*), vascular cell adhesion molecule 1 (*Vcam1*), and kinase insert domain receptor (*Kdr*) were significantly increased in 1 or 10 mM genipin condition compared to 0.01 mM (Fig. 3B). The expression of endothelial inflammatory cytokines, *Il-6*, *Ccl2*, *Ccl4*, and *Ccl20*, were increased in 0.1, 0.5, 1, and 10 mM genipin (Fig. 3B). Time course analysis showed that 12 h–48 h incubation did not affect *Ccl2* and *Ccl20* on 0.05, 0.1, and 0.5 mM genipin gels (Fig. 3C).

To test the possibility that genipin may be an inflammatory inducer, we stimulated adipose tissue component cells with 0.01–10 mM genipin solution. There were no effects of genipin stimulation on differentiation- and inflammation-related genes including *Pparg2*, *Il-6*, and *Cxcl13* in adipocytes (supplemental Fig. S2A), *Irf5* and *Ifng* in M1 macrophages (supplemental Fig. S2B), *Nos2* and *Il-10* in M2 macrophages (supplemental Fig. S2C), and *Tek* and *Ccl2* in endothelial cells (supplemental Fig. S2D).

Collectively, these results indicate that matrix stiffness regulates the variety of gene expression in adipose tissue component cells including adipocytes, macrophages, and endothelial cells. Hard matrixes, which prepared with 0.5–10 mM genipin cross-linked collagen, increased inflammatory gene expression in adipocytes, M1 macrophages, and endothelial cells. Conversely, soft matrixes, which were prepared with 0.01 and 0.05 mM genipin cross-linked collagen, enhanced anti-inflammatory gene expression in adipocytes and M2 macrophages. In 0.01–10 mM genipin-treated collagen gels, the 0.1 mM condition seems to be the central value for distinguishing between the inflammatory state and the antiinflammatory state.

### The effects of matrix stiffness on adipocytes, macrophages, and endothelial cells are transmitted between each cell type through the secretory factors

Previous studies suggest that the stiffness of adipose tissue varies among different regions due to unequal lipid droplet size, macrophage infiltration, and vascular distribution (22, 23). Because adipocytes, macrophages, and endothelial cells respond to the matrix stiffness and highly produce a variety of cytokines (Fig. 1, Fig. 2, Fig. 3), we hypothesized that the effects of matrix stiffness on the one component cells of adipose tissue are transmitted to the other component cells.

**Fig. 4:**
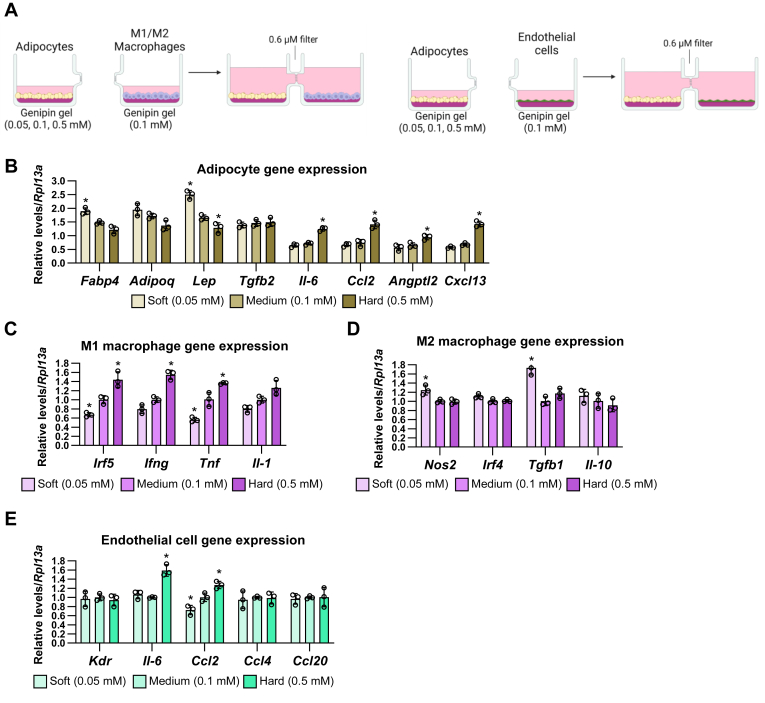
The effects of matrix stiffness on adipocytes are transmitted to the distant M1/M2 macrophages and endothelial cells through the secretory factors. A: Schematic illustration of coculture of adipocytes under different stiffness with M1/M2 macrophages or endothelial cells under normal stiffness. B-E: The mRNA expression of adipocytes (B), M1 macrophages (C), M2 macrophages (D), and endothelial cells (E) under the coculture of matrix stiffness-stimulated adipocytes. (∗*P* < 0.01 vs. 0.1 mM with one-way ANOVA and Tukey and Bonferroni post hoc analysis).

First, we stimulated mature adipocytes in the culture units coated with three types of genipin-crosslinked gels: soft matrix: 0.05 mM genipin, medium matrix: 0.1 mM genipin, and hard matrix: 0.5 mM genipin. Separately, M1 macrophages, M2 macrophages, and endothelial cells were also stimulated in the other culture units coated with 0.1 mM genipin gel. After 24 h incubation, the culture units were connected to each other, and adipocytes and three types of cells were cocultured in shared media under different matrix stiffness conditions for 24 h (Fig. 4A). Similar to the one-unit results (Fig. 1), soft matrix stiffness increased mRNA levels of *Fabp4* and *Lep* and hard matrix stiffness increased mRNA levels of *Il-6*, *Ccl2*, and *Cxcl13* and decreased *Lep* expression in adipocytes (Fig. 4B). In M1 macrophages, compared to the coculture with adipocyte on the medium matrix, the coculture with adipocytes on the soft matrix significantly decreased the expression of *Irf5* and *Tnf*, whereas the coculture of adipocyte on the hard matrix increased the expression of *Irf5*, *Ifng*, and *Tnf* (Fig. 4C). In M2 macrophages, the coculture with adipocytes on the soft matrix significantly increased the expression of *Nos2* and *Tgfb1*; however, the coculture of adipocyte on the hard matrix did not affect M2 phenotypic gene expression (Fig. 4D). In endothelial cells, the coculture with adipocytes on the soft matrix significantly decreased the expression of *Ccl2*, whereas the coculture of adipocyte on the hard matrix increased the expression of *Il-6* and *Ccl2* (Fig. 4E). These results suggest that the effects of matrix stiffness on adipocytes affect the inflammatory gene expression in the distant M1/M2 macrophages and endothelial cells through the secretory factors.

**Fig. 5:**
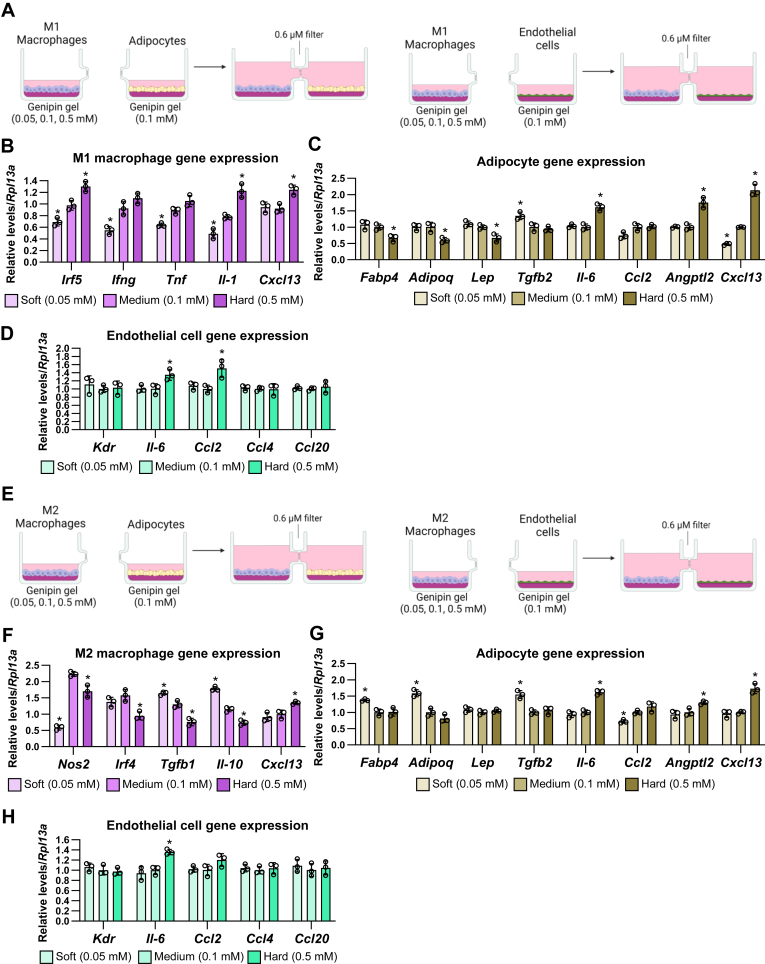
The effects of matrix stiffness on M1/M2 macrophages are transmitted to the distant adipocytes and endothelial cells through the secretory factors. A and E: Schematic illustration of coculture of M1 (A) or M2 (E) macrophages under different stiffness with adipocytes or endothelial cells under normal stiffness. B-D and F-H, The mRNA expression of M1 macrophages (B), M2 macrophage (F), adipocyte (C and G), and endothelial cells (D and H) under the coculture of matrix stiffness-stimulated M1 (B–D) or M2 (F–H) macrophages. (∗*P* < 0.01 vs. 0.1 mM with one-way ANOVA and Tukey and Bonferroni post hoc analysis).

To examine whether the effects of matrix stiffness on M1 macrophages are similarly transmitted to the distant adipocytes or endothelial cells in shared culture medium, we cocultured mature adipocytes and endothelial cells on the medium matrix with M1 macrophages on the soft, medium, and hard genipin gels for 24 h (Fig. 5A). In these culture units, the soft stiffness deceased mRNA levels of *Irf5*, *Ifng*, *Tnf*, and *Il-1* and the hard stiffness increased mRNA levels of *Irf5*, *Il-1*, and *Cxcl13* (Fig. 5B). In the case of the coculture of mature adipocytes with M1 macrophages, the soft matrix significantly increased *Tgbfb2* mRNA expression and decreased *Cxcl13* mRNA expression in adipocytes, whereas the hard matrix increased the expression of *Il-6*, *Angptl2*, and *Cxcl13* and decreased the expression of *Fabp4*, *Adipoq*, and *Lep* (Fig. 5C). In the case of the coculture of endothelial cells, the hard matrix increased the expression of *Il-6* and *Ccl2*, whereas the soft matrix did not affect the endothelial inflammatory gene expression (Fig. 5D). We also cocultured mature adipocytes and endothelial cells on the medium matrix with M2 macrophages on the soft, medium, and hard genipin gels for 24 h (Fig. 5E). In these culture units, the soft stiffness decreased *Nos2* expression and increased *Tgfb1* and *Il-10* expression (Fig. 5F). The hard stiffness decreased *Nos2*, *Irf4*, *Tgfb1*, and *Il-10* expression and increased *Cxcl13* expression. In the case of the coculture of mature adipocytes with M2 macrophages, the soft matrix significantly increased *Fabp4*, *Adipoq*, and *Tgbfb2* mRNA expression and decreased *Ccl2* mRNA expression in adipocytes, whereas the hard matrix increased the expression of *Il-6*, *Angptl2*, and *Cxcl13* (Fig. 5G). In the case of the coculture of endothelial cells, the hard matrix increased the expression of *Il-6* (Fig. 5H). Similar to the transmission of the effects of matrix stiffness from adipocytes to other cells, these results indicate that the effects of matrix stiffness on M1/M2 macrophages induce inflammation-related gene expression in distant adipocytes and endothelial cells through bioactive molecules.

**Fig. 6:**
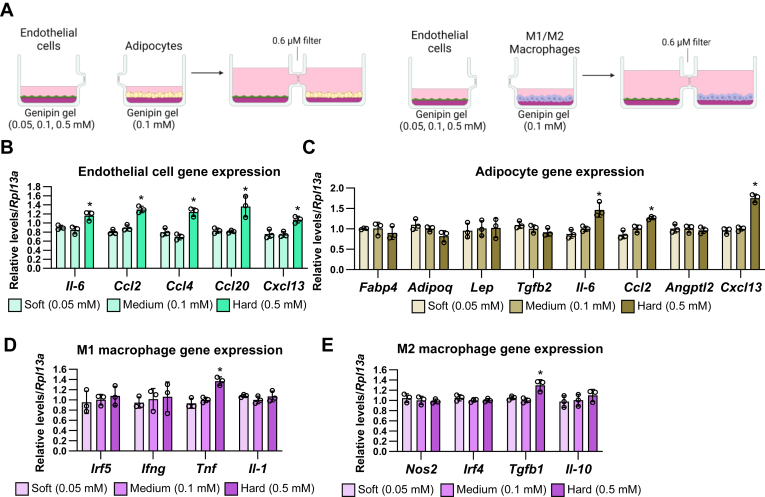
The effects of matrix stiffness on endothelial cells are transmitted to the distant adipocytes and M1/M2 macrophages through the secretory factors. A: Schematic illustration of coculture of endothelial cells under different stiffness with adipocytes or M1/M2 macrophages under normal stiffness. B–E: The mRNA expression of endothelial cells (B), adipocytes (C), M1 macrophages (D), and M2 macrophages (E) under the coculture of matrix stiffness-stimulated endothelial cells. (∗*P* < 0.01 vs. 0.1 mM with one-way ANOVA and Tukey and Bonferroni post hoc analysis).

Moreover, we cocultured the soft, medium, or hard matrix-stimulated endothelial cells with the differentiated adipocytes and the endothelial cells (Fig. 6A). In these culture units, the hard stiffness increased mRNA levels of *Il-6*, *Ccl2*, *Ccl4*, *Ccl20*, and *Cxcl13* in endothelial cells (Fig. 6B). In the case of the adipocytes, the soft matrix did not change the expression of adipokines and inflammatory cytokines, whereas the hard matrix increased the expression of *Il-6* and *Cxcl13* (Fig. 6C). M1 and M2 macrophages also did not respond to the effects of soft matrix on endothelial cells; however, the hard matrix increased *Tnf* expression in M1 macrophages and *Tgfb1* expression in M2 macrophages (Fig. 6D, E). Collectively, these results indicate that adipocytes, macrophages, and endothelial cells transmit their matrix stiffness information into the distant cells and change the state of inflammation in the adipose tissue component cells.

### CXCL13 is one of the signaling proteins of matrix stiffness among adipocytes, macrophages, and endothelial cells

**Fig. 7:**
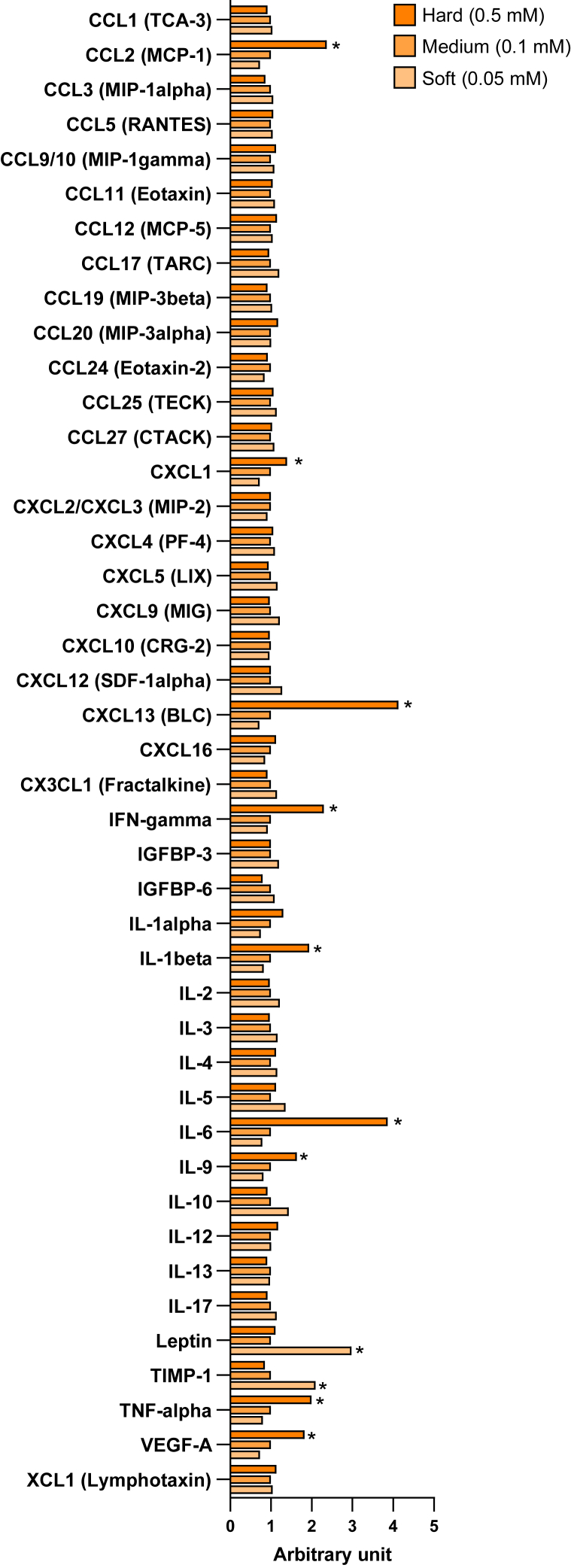
Hard matrix stiffness induces the secretion of inflammatory cytokines from adipocytes. The relative detection levels of inflammatory cytokines in the culture media of adipocytes under the stimulation of different stiffness. (∗*P* < 0.01 vs. 0.1 mM with one-way ANOVA and Tukey and Bonferroni post hoc analysis).

Our coculture models suggest that the matrix stiffness-stimulated cells secret the cytokines that can affect the expression of inflammatory genes in distant cells. Because previous studies reported that the changes of ECM stiffness initially occurred in the fibroblastic tissues near the adipocytes in adipose tissue (24, 25), we hypothesize that the effects of matrix stiffness on inflammatory gene expression in the adipose tissue component cells initially occur from adipocyte-derived secretory factors. We cultured the mature adipocytes on 0.05 mM (soft), 0.1 mM (medium), and 0.5 mM (hard) genipin-treated cross-linked collagen gels for 24 h and performed the protein array analysis of the conditioned media (Fig. 7). The soft matrix increased the levels of leptin and tissue inhibitor of metalloproteinase-1 (TIMP-1) by more than 1.5-fold and decreased the levels of CCL2, CXCL1, CXCL13, IL-1α, IL-6, TNF-α, and vascular endothelial growth factor-A (VEGF-A) by less than 0.7-fold in conditioned media. On the other hand, the hard matrix increased the levels of CCL2, CXCL13, IFN-γ, IL-1β, IL-6, IL-9, TNF-α, and VEGF-A by more than 1.5-fold.

**Fig. 8:**
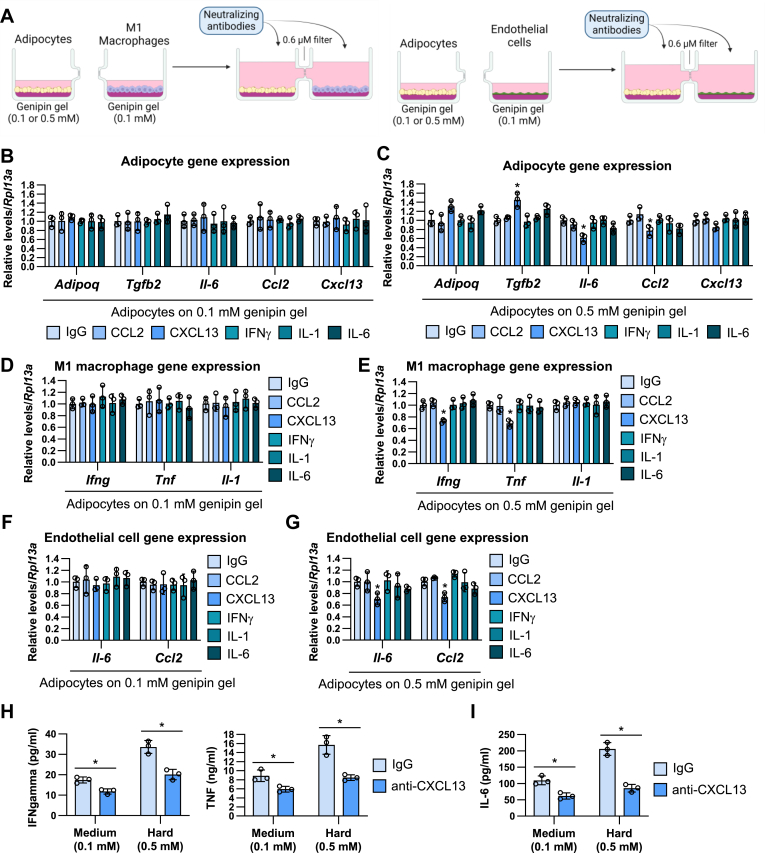
The transmission of the effects of matrix stiffness on adipocytes into the distant cells is suppressed by CXCL13 neutralizing antibodies. A: Schematic illustration of coculture of adipocytes under the medium (0.1 mM genipin) or hard (0.5 mM genipin) stiffness with M1 macrophages or endothelial cells under the medium stiffness with the treatment of neutralizing antibodies. B–G: The mRNA expression of adipocytes (B and C), M1 macrophages (D and E), and endothelial cells (F and G) under the coculture of the medium (B, D, and F) or hard (C, E, and G) matrix stiffness-stimulated adipocytes with neutralizing antibodies. (∗*P* < 0.01 vs. IgG with one-way ANOVA and Tukey and Bonferroni post hoc analysis). H and I: The amount of IFN-γ, TNF (H), and IL-6 (I) in the culture media of M1 macrophage (H) or endothelial cells (I) under the co-culture of the medium or hard matrix stiffness-stimulated adipocytes. (∗*P* < 0.01 vs. IgG with two-way ANOVA and Tukey and Bonferroni post hoc analysis). IFN-γ, interferon-gamma; IgG, immunoglobulin G; IL-6, interleukin-6; TNF, tumor necrosis factor.

Based on this secretory protein array data and the mRNA expression profile of matrix-stimulated adipocytes (Fig. 2), we selected CCL2, CXCL13, IFN-γ, IL-1, and IL-6 as the candidate proteins that can transmit the matrix stiffness information to M1/M2 macrophages and endothelial cells and promote the inflammatory gene expression. We cocultured mature adipocytes on the medium (0.1 mM genipin) or hard matrix (0.5 mM genipin) with M1 macrophages and endothelial cells on the medium matrix for 24 h in the addition of the neutralizing antibodies against CCL2, CXCL13, IFN-γ, IL-1, and IL-6 (Fig. 8A). In coculture with the medium stiffness-stimulated adipocytes, the inflammatory phenotypes were not changed by these antibody treatments in the adipocytes, M1 macrophages, and endothelial cells (Fig. 8B, D, F). In contrast, anti-CXCL13 antibody treatment increased the expression of *Tgfb2* and decreased the expression of *Il6* and *Ccl2* in the hard matrix-stimulated adipocytes (Fig. 8C). Similarly, the anti-CXCL13 antibody blocked *Ifn5* and *Tnf* expression in M1 macrophages (Fig. 8E) and *Il6* and *Ccl2* expression in endothelial cells (Fig. 8G) under the coculture with hard matrix-stimulated adipocytes. The productions of inflammatory cytokines such as IFNγ and TNF in M1 macrophages (Fig. 8H) and IL-6 in endothelial cells (Fig. 8I) were also blocked by anti-CXCL13 antibody treatments under both the coculture with medium and hard matrix stiffness-stimulated adipocytes.

**Fig. 9:**
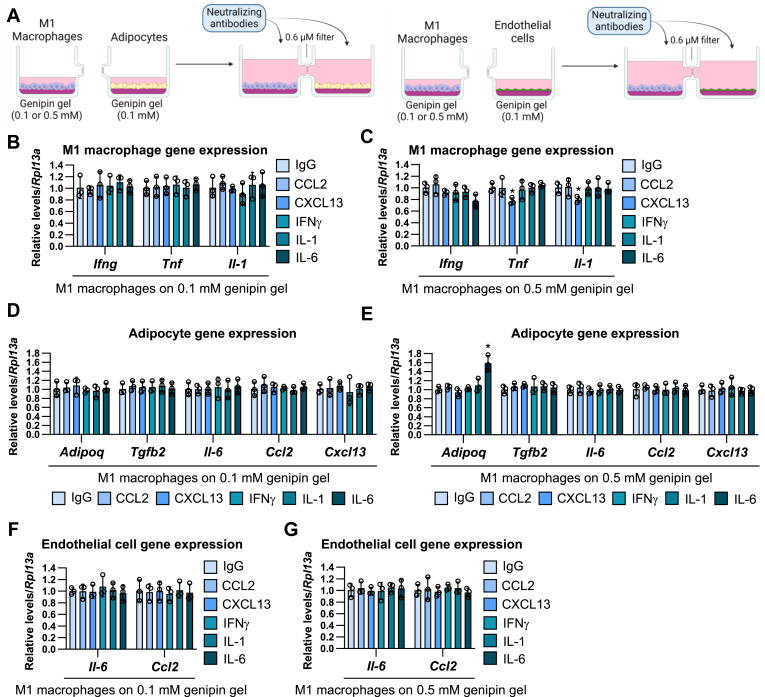
The transmission of the effects of matrix stiffness on M1 macrophages into the distant cells is not affected by CXCL13 neutralizing antibodies. A: Schematic illustration of coculture of M1 macrophages under the medium (0.1 mM genipin) or hard (0.5 mM genipin) stiffness with adipocytes or endothelial cells under the medium stiffness with the treatment of neutralizing antibodies. B-G: The mRNA expression of M1 macrophages (B and C), adipocytes (D and E), and endothelial cells (F and G) under the coculture of the medium (B, D, and F) or hard (C, E, and G) matrix stiffness-stimulated M1 macrophages with neutralizing antibodies. (∗*P* < 0.01 vs. IgG with one-way ANOVA and Tukey and Bonferroni post hoc analysis). IgG, immunoglobulin G.

To examine the effects of M1 macrophage-derived cytokines on inflammatory gene expression in adipocytes and endothelial cells, we further cocultured M1 macrophages on the medium or hard matrix with adipocytes and endothelial cells on the medium matrix for 24 h in the addition of the neutralizing antibodies against CCL2, CXCL13, IFN-γ, IL-1, and IL-6 (Fig. 9A). In coculture with the medium stiffness-stimulated adipocytes, the inflammatory phenotypes were not changed by these antibody treatments in the adipocytes, M1 macrophages, and endothelial cells (Fig. 9B, D, F). Similar to the coculture with hard matrix-stimulated adipocytes, the treatment of anti-CXCL13 antibody blocked *Tnf* and *Il-1* expression in hard matrix-stimulated M1 macrophages (Fig. 9C). However, there were no effects of CXCL13 blocking on the inflammatory gene expression in cocultured adipocytes (Fig. 9E) and endothelial cells (Fig. 9G). The treatment of anti-IL6 antibody specifically increased *Adipoq* expression in adipocytes (Fig. 9E).

**Fig. 10:**
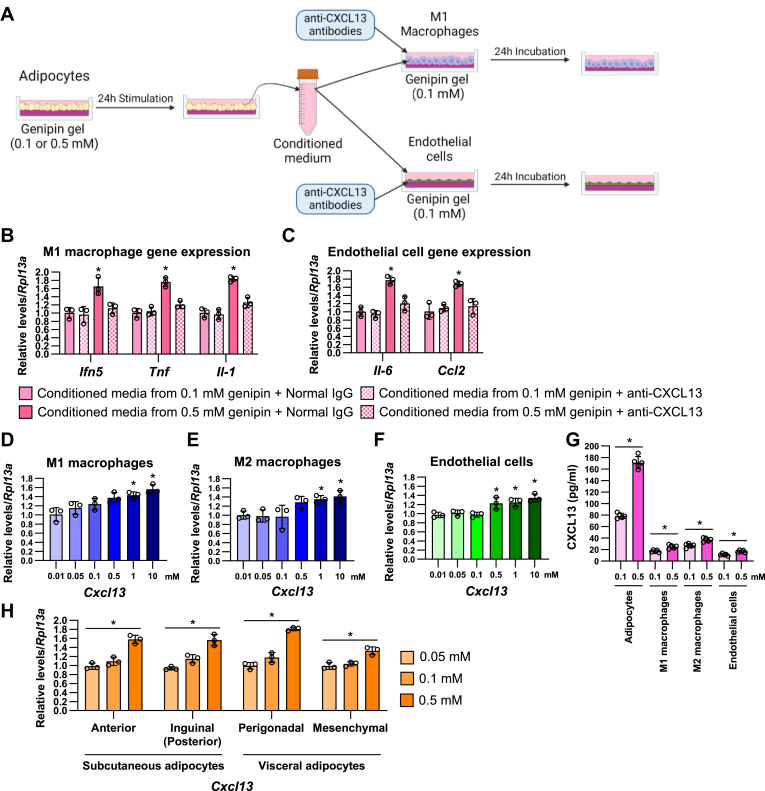
Adipocytes are the main source of CXCL13 under the hard stiffness conditions. A: Schematic illustration of conditioned media treatment from the matrix stiffness-stimulated adipocytes with M1 macrophages or endothelial cells. B and C: The mRNA expression of M1 macrophages (B) or endothelial cells (C) under the treatment of the conditioned media from adipocytes in the addition of anti-CXCL13 antibodies for 24 h. (∗*P* < 0.01 vs. conditioned media from 0.1 mM genipin + normal IgG with two-way ANOVA and Tukey and Bonferroni post hoc analysis). D-F: The *Cxcl13* mRNA expression of M1 macrophages (D), M2 macrophages (E), and endothelial cells (F) under the stimulation with the different types of matrix stiffness for 24 h. (∗*P* < 0.01 vs. 0.1 mM) G: The amount of CXCL13 protein in cell culture media from adipocytes, M1 macrophages, M2 macrophages, and endothelial cells that were stimulated by 0.1 or 0.5 genipin-crosslinked gel for 24 h. (∗*P* < 0.01 vs. 0.1 mM with one-way ANOVA and Tukey and Bonferroni post hoc analysis) H: The *Cxcl13* mRNA expression of adipocytes from anterior or posterior subcutaneous and perigonadal or mesenchymal visceral adipose tissues under the stimulation with 0.1 or 0.5 mM genipin-crosslinked gels for 24 h. (∗*P* < 0.01 vs. 0.1 mM with one-way ANOVA and Tukey and Bonferroni post hoc analysis).

Furthermore, we collected the conditioned media from 0.1 mM (medium) or 0.5 mM (hard) genipin-crosslinked gel-stimulated adipocytes and stimulated M1 macrophages and endothelial cells with 50% conditioned media in the addition of anti-CXCL13 antibodies (Fig. 10A). The treatment of the conditioned media from the medium gel-stimulated adipocytes did not affect inflammatory gene expression in M1 macrophages and endothelial cells (Fig. 10B, C). In contrast, the conditioned media from the hard gel-stimulated adipocytes increased mRNA levels of *Ifn5*, *Tnf*, and *Il-1* in M1 macrophages and *Il-6* and *Ccl2* in endothelial cells and these effects were completely reversed by anti-CXCL13 antibody treatment.

To explore the source of CXCL13 in the different stiffness conditions, we analyzed *Cxcl13* mRNA levels in M1 macrophages (Fig. 10D), M2 macrophages (Fig. 10E), and endothelial cells (Fig. 10F) under the culture of 0.01, 0.05, 0.1, 0.5, 1, and 10 mM genipin-crosslinked gels. Similar to the adipocytes (Fig. 1C), Cxcl13 expressions were gradually increased from 0.5 to 10 mM genipin among these cells. We further analyzed the amount of CXCL13 proteins from the culture media of 0.1 or 0.5 mM genipin gel-stimulated adipocytes, M1 macrophages, M2 macrophages, and endothelial cells. CXCL13 was predominantly produced from adipocytes compared to the other adipose tissue component cells and was highly induced by 0.5 mM genipin conditions (Fig. 10G). Finally, we isolated different types of adipocytes from subcutaneous (anterior and inguinal (posterior) parts) and visceral (perigonadal and mesenchymal parts) adipose tissues respectively, and these adipocytes were stimulated 0.05, 0.1, and 0.5 mM genipin-crosslinked gels for 24 h (Fig. 10H). Hard matrix-induced *Cxcl13* expression was increased in all types of adipocytes. Collectively, these results suggest that CXCL13 is an adipocyte-derived inflammatory cytokine that transmits the matrix stiffness information of the adipocyte to adipose tissue component cells.

## Discussion

The changes of ECM mechanical properties affect the adipose tissue component cells through mechanotransduction signaling. Inflammation and fibrosis are crucial exogenous factors to dysregulate adipose tissue functions and drastically changes the mechanical properties of ECM (7, 8). Therefore, the crosstalk communication among ECM and adipose tissue component cells is necessary to understand the multifaced functions of adipose tissues such as energy storage, glucose and fatty acid metabolism, and endocrines. Here, we found that the matrix stiffness regulates the gene expression of primary adipocytes, M1/M2 macrophages, and endothelial cells. Among these three types of cells, the direction of inflammatory states and antiinflammatory states had the same matrix stiffness in vitro. The information of matrix stiffness-stimulated adipocytes was transmitted to the distant macrophages and endothelial cells through adipocyte-derived secretory factors. We discovered that hard matrix induces the expression of *Cxcl13* in adipocytes, and CXCL13 is one of the important candidate proteins to communicate with macrophages and endothelial cells for sharing matrix stiffness information.

ECM stiffness is known to influence inflammatory states through mechanotransduction axis. For example, the excessive deposition of ECM-induced matrix stiffness raises the inflammatory responses in the progression of fibrosis (26). Increased ECM stiffness also primed vascular smooth muscle cells to a proinflammatory phenotype (27). Conversely, human gingival fibroblasts are exacerbated by the proinflammatory responses in soft substrate culture (28), suggesting that each cell type may have the specific stiffness to show proinflammatory or antiinflammatory conditions. Previous studies showed that adipose tissue stiffness is associated with adipogenic differentiation and obesity-related expansion of adipocytes with lipid droplet accumulation; however, there are controversial reports regarding the relationship between mechanical alterations and the induction of adipogenic differentiation in mesenchymal stem cells, adipose-derived stem cells, and adipo-progenitor cells (15, 29, 30, 31, 32). Furthermore, while the stiffness-induced secretion of VEGF in adipose-derived stromal cells and adipose-derived mesenchymal stem cells has been studied (33, 34), the effects of matrix stiffness on adipocyte-derived secretory factors remain unelucidated. Our study revealed that mature adipocytes produce adipogenic secretory factors, inflammatory cytokines, and chemokines (Figs. 1 and 7), suggesting that ECM deposition comprehensively affects the endocrine functions of adipocytes during differentiation.

Previous studies showed that macrophages and endothelial cells are typical matrix stiffness-responsive cells. For example, matrix stiffness is an important factor in regulating M1/M2 polarization (35, 36, 37, 38, 39, 40), migration (38, 41), and inflammatory cytokine production (41, 42) in macrophages. Angiogenic proliferation (43, 44, 45), VEGF signaling (46), endothelial barrier integrity (47), and vasodilation (48) are also affected by matrix stiffness in endothelial cells. Similarly, our genipin-manipulated collagen gel models showed the stiffness-regulated gene expression in primary M1/M2 macrophages and endothelial cells (Figs. 2 and 3). One of our most interesting findings is that these matrix-stiffness-induced changes are induced by the coculture of hard matrix-stimulated adipocyte-derived CXCL13 in both macrophages and endothelial cells (Figs. 4, 5 and 8). Previous studies reported other examples of stiffness-activated paracrine signaling in several types of cell combinations such as endothelial cell proliferation via cardiomyocyte-derived VEGF (49), osteogenic differentiation of bone marrow-derived mesenchymal stem cells by macrophages (50), macrophage migration and polarization via breast cancer cell-derived colony stimulating factor 1 (38), and inflammatory reaction of macrophages via mesenchymal stem cell-derived tumor necrosis factor-α-stimulated protein 6 (51). These studies suggest that matrix stiffness is regarded as information that can induce pathophysiological changes in distant cells. In adipose tissue, adipocytes are the main governors of matrix stiffness responses by the high production of CXCL13 under hard matrix stiffness. Cxcl13 expression in adipocytes was also increased by the coculture with hard matrix-stimulated M1/M2 macrophages (Fig. 5C, G) and endothelial cells (Fig. 6C). We hypothesize that CXCL13 is used to transmit the stiffness information in all adipose tissue area, and adipocyte amplify matrix stiffness changes by high production of CXCL13 among adipose tissue component cells. We found that CXCL13 was predominantly produced from adipocytes compared to the other adipose tissue component cells and was highly induced by hard stiffness (0.5 mM genipin) conditions (Fig. 10G). Based on these data, we propose that adipocytes, a main component of adipose tissues, mainly respond to matrix stiffness and transmit this information to macrophages and endothelial cells through CXCL13. Macrophages and endothelial cells also respond to matrix stiffness and increase the levels of CXCL13 in adipocytes, leading to the enhancement of the responses to matrix stiffness information among adipose tissue component cells. Collectively, matrix stiffness information may be shared with triad patterns of adipocytes, macrophages, and endothelial cells, and CXCL13 is one of the central regulators of this system.

We previously reported that CXCL13 is a differentiation- and hypoxia-induced adipocytokine that induces the inflammatory phenotypes of adipocytes (52). Excessive hypertrophy of adipocytes and over-deposition of ECM is a typical feature of hypoxic conditions in the adipose tissue (53). Therefore, CXCL13 may integrate the detrimental effects of obesity-induced pathological events on adipose tissue function. It is currently difficult to diagnose the stiffness of adipose tissue in clinical situations. Since recent studies utilize the plasma levels of CXCL13 as a biomarker to predict disease activity (54, 55), adipose tissue stiffness-induced changes may be evaluated by serum or plasma levels of CXCL13. Additionally, CXCL13 works as the transmitter of matrix stiffness information from the adipocytes under the hard area to adipose tissue component cells under the soft area. CXCL13 blocking may become a new therapeutic target that can block the hard matrix area-initiated inflammatory reaction.

There are several technical limitations in our study. First, we isolated primary macrophages by intraperitoneal injection of thioglycolate. These macrophage phenotypes may have different characteristics compared to adipose tissue-resident macrophages. Second, we cultured the multilocular adipocytes in vitro, and these cells may have different mechanical properties compared to uniocular adipocytes in vivo. Third, since we have performed the cytokine array but not comprehensive proteomics in the culture media from matrix stiffness-stimulated adipocytes and the treatment of CXCL13 neutralizing could not completely block the transmission of matrix stiffness, there may exist the potential transmitters of stiffness information.

In conclusion, we have determined the cellular physiological mechanisms underlying the effects of matrix stiffness to regulate the inflammatory phenotypes on the adipose tissue component cells including adipocytes, macrophages, and endothelial cells. A hard matrix induces the inflammatory gene expression in all three types of cells, whereas a soft matrix induces the antiinflammatory gene expression. The effects of matrix stiffness on inflammatory phenotypes in each cell type are transmitted to the distant cells that were incubated under different stiffness conditions. Among the secretory proteins that derived from adipocytes stimulated by different stiffness, CXCL13 is the most important transmitter of hard matrix information that causes inflammation to distant macrophages and endothelial cells. These findings may provide insight into the application of stiffness control for chronic inflammation in adipose tissues with metabolic dysregulation.

## Data Availability

There are neither new datasets/codes generated nor restrictions for use of the materials in this article. Further information and requests for resources and reagents should be directed to and will be fulfilled by the Lead Contact, Joji Kusuyama (joji.kusuyama.bsin@tmd.ac.jp).

## Supplemental data

This article contains supplemental data.

## Conflict of interest

The authors declare that they have no conflicts of interest with the contents of this article.

## Supplemental Data

Supplemental Figure 1 Adipogenic differentiation of primary adipocytes on the genipin-crosslinked gel. Oil Red O staining of day 0 and day 8 primary adipocytes. Scale bar: 100 μm.

Supplemental Figure 2 The effects of genipin solution on differentiation- and inflammation-related gene expression in adipose tissue component cells. A–D. Differentiation- and inflammation-related gene expression in adipocytes (A), M1 macrophages (B), M2 macrophages (C), and endothelial cells (D) under the stimulation of 0 to 10 mM genipin solution for 24 h.

Supplemental Table 1 List of primer sequences used in realtime quantitative PCR.
