# Supplementary figures and images for: Matrix stiffness regulates the triad communication of adipocytes/macrophages/endothelial cells through CXCL13
Source: J Lipid Res. 2024 Aug 14;65(9):100620. doi: 10.1016/j.jlr.2024.100620 (PMC11406362; doi:10.1016/j.jlr.2024.100620)

**Day 0**

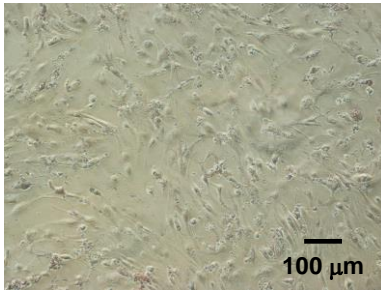

**Day 8**

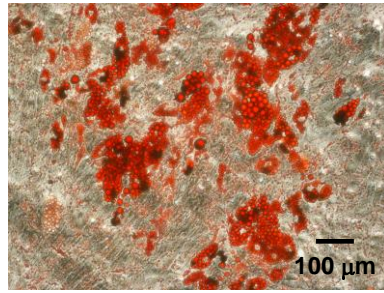

Supplement: Supplemental Figure 2 — The effects of genipin solution on differentiation- and inflammation-related gene expression in adipose tissue component cells. A–D. Differentiation- and inflammation-related gene expression in adipocytes (A), M1 macrophages (B), M2 macrophages (C), and endothelial cells (D) under the stimulation of 0 to 10 mM genipin solution for 24 h. [file mmc2.pdf]

A

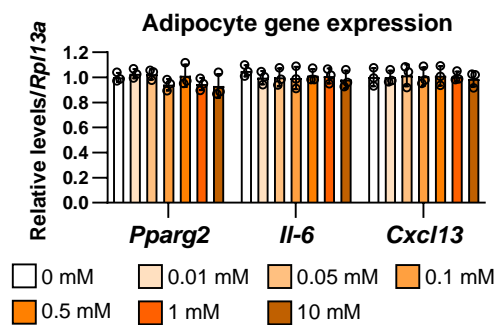

B

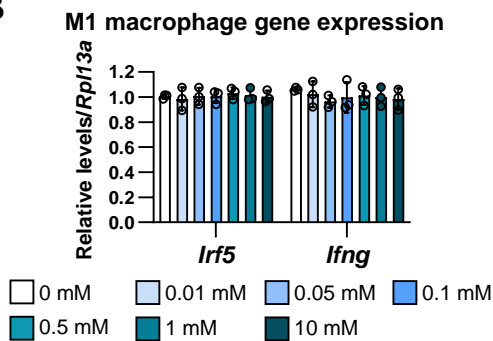

C

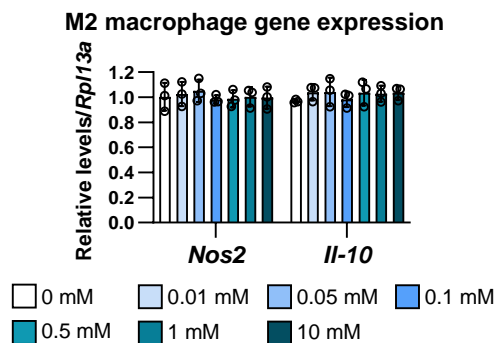

D

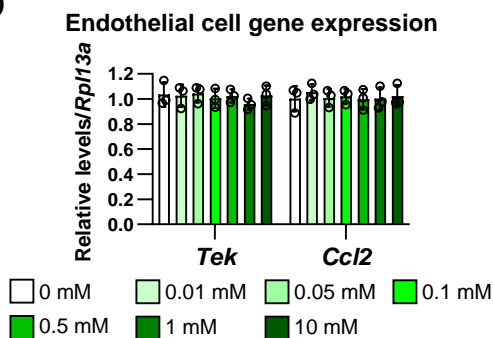

Supplement: Supplemental Table 1 — List of primer sequences used in realtime quantitative PCR. [file mmc3.pdf]
